# Associations between Time Processing Ability, Daily Time Management, and Dementia Severity

**DOI:** 10.3390/ijerph19073928

**Published:** 2022-03-25

**Authors:** Ann-Christine Persson, Gunnel Janeslätt, Lena Dahlberg, Monika Löfgren, Marika Möller

**Affiliations:** 1 Department of Clinical Sciences, Danderyd Hospital, Karolinska Institutet, 18288 Stockholm, Sweden; monika.lofgren@ki.se (M.L.); marika.moller@ki.se (M.M.); 2 Department of Rehabilitation Medicine, Danderyd University Hospital, 18288 Stockholm, Sweden; 3 Department of Public Health and Caring Sciences, Uppsala University, 75105 Uppsala, Sweden; gunnel.janeslatt@pubcare.uu.se or; 4 Center for Clinical Research in Dalarna, Box 712, 79129 Falun, Sweden; 5 School of Health and Welfare, Dalarna University, 79188 Falun, Sweden; ldh@du.se; 6 Aging Research Center, Karolinska Institutet & Stockholm University, 17165 Solna, Sweden

**Keywords:** Alzheimer’s disease, attention, cognition, elderly, memory assessment, MMSE, time management, time orientation, time perception, visuospatial

## Abstract

This study investigated associations between time processing ability (TPA), daily time management (DTM), and dementia severity. Persons with dementia (PwDs) (*n* = 53) and their significant others (*n* = 49) participated in this cross-sectional study. Bivariate analyses were used to investigate associations between TPA and DTM and the dementia severity. Linear regression models were used to further predict the contribution of the subtests in the Mini Mental State Examination (MMSE) for TPA results. The results showed significant correlations between TPA and dementia severity, where visuospatial functions were the most highly correlated. TPA also showed a significant correlation to proxy-rated DTM. In addition, proxy-rated DTM was significantly correlated with dementia severity and PwDs’ own self-ratings of their DTM. Knowledge of the association between TPA, dementia severity, and visuospatial functions can enable early detection of TPA impairments. For a comprehensive assessment of TPA and DTM, objective measures should be used in combination with self-ratings and proxy-ratings. The findings can be used in clinical research and healthcare settings to develop methods to compensate for impaired TPA and support DTM in PwDs.

## 1. Introduction

Dementia is characterised by a progressive deterioration in cognition, affecting, among other things, memory and visuospatial functions [1], time perception and time orientation [2,3], and the ability to manage time [4]. Persons with dementia (PwDs) often need frequent support in daily time management (DTM) by significant others or formal caregivers, for example, to keep track of time, to arrive on time for scheduled activities, and to plan activities in a time-specific order with enough time allocated [5,6,7].

In the International Classification of Functioning, Disability, and Health (ICF) [8], time-related concepts are described at both the body function and activity and participation domains. Orientation to time, experience of time, and time management are included in the body function domain. Orientation to time includes both time concepts, such as dates, and objective time, such as, for example, understanding and using the clock or other devices for orientation in time. Experience of time includes knowledge of time duration. Time management is part of executive functions and encompasses knowing when to undertake an activity, for how long, and allocating time for different activities. These functions together form the hierarchical construct of time processing ability (TPA) [9,10]. DTM, on the other hand, is part of several components in the category “carrying out a daily routine” in the activity and participation domain, which illustrates the complexity of DTM [8,11]. DTM requires, for example, the ability to plan for and carry out daily activities in a time-specific order within requested time limits, and, if necessary, to adjust for unforeseen events. Thus, DTM has a central role in the everyday functioning.

Though dementia can affect both time-related cognitive functions and time-related occupations in daily life, in-depth assessments of TPA and DTM for PwDs are limited [7]. Until now, there have been a lack of validated instruments assessing all three levels of TPA and DTM for PwDs [12], although common assessments such as the Clock Drawing Test and Mini Mental State Examination (MMSE) [13] include aspects of the ability to understand and orient oneself in time and space. Previous research on children with and without neurodevelopmental diagnoses has found that TPA is related to parent-rated DTM [14] and, in youth, there is a low but non-significant correlation with self-rated DTM [15], but there is a lack of knowledge if this is also valid for PwDs. Such knowledge would increase understanding on how assessments of TPA and DTM in PwDs should be interpreted in relation to each other.

Furthermore, to be able to provide appropriate support at the right time for PwDs, it is also important to enhance the understanding of how TPA and DTM are related to the progress of cognitive deficits in dementia. Previous research has found that orientation to time is related to the dementia severity [16,17,18], a relationship that might be linked to the hippocampus, which processes both spatial and temporal information [19]. Changes in the hippocampus and frontal lobes affecting spatial functions have also been connected to time perception impairments at an early stage in persons with Alzheimer’s disease [2,20]. However, orientation to time is just one component of TPA. Further research about the relation between dementia severity and all three levels of TPA can promote the development of assessment methods in healthcare services. Early detection of time-related impairments can increase the possibility that interventions—such as time assistive technology or compensatory strategies—can be implemented in a phase when cognitive abilities are relatively preserved and PwDs have more resources available to adopt new courses of action [7]. Examples of time assistive technology are devices that support time orientation by displaying time, day and date, or electronic calendars with information about and reminders for important activities. Compensatory strategies in DTM could be to keep a calendar in a visible place in the home to be able and see and refer to it frequently, to get ready in advance by gathering items that need to be brought to an appointment, or to use a direct debit to pay bills on time. Therefore, increased understanding of the relationships between TPA and DTM and dementia severity is essential, because it is a cornerstone in clinical assessments and future planning of intervention studies. The aim of this study was to investigate the associations between TPA, DTM, and dementia severity.

## 2. Materials and Methods

This study has a cross-sectional, explorative design and is part of the intervention study “Managing Time with Dementia” project. A study protocol is registered at www.clinicaltrials.gov, accessed on 8 March 2022, reg. no. NCT03677284. The study was approved by the Regional Ethical Review Board in Uppsala (reg. no. 2018/059, approved 16 May 2018).

### 2.1. Participants

PwDs (*n* = 53) and significant others (SOs; *n* = 49), such as spouses and children, were included (Table 1). The inclusion criteria for PwDs were a dementia diagnosis established by nationally established methods at the memory clinics, ≥11 on the Mini-Mental State Examination (MMSE) [13,21], problems with DTM identified by occupational therapists through memory assessments, age ≥ 60 years, ability to communicate in Swedish, and no mental illness unrelated to dementia. The inclusion criteria for SOs were knowledge about the PwD’s daily life and DTM, and the ability to communicate in Swedish.

### 2.2. Materials

#### 2.2.1. Demographic Items

Demographic data were collected via study-specific questionnaires including questions about gender, age, dementia diagnosis, MMSE, family situation (single, living alone; partner, not living together; partner/family, living together), and education (elementary school, high school, or college/university) for PwDs, and questions about gender, age, relation to participant (spouse, child), and support given days/week (≤1; 2–3; daily) for SOs.

#### 2.2.2. Assessment to Measure TPA

KaTid^®^ is a standardised and validated test to measure TPA in persons with cognitive impairment [8,9,14,22,23,24]. KaTid was originally developed for children and youths [9]. A new version, KaTid-Senior, has been developed for persons with mild to moderate dementia. The test consists of 26 items across four subcategories: (a) time perception/time sense (3 items), (b) time orientation/time concepts (8 items), (c) time orientation/objective time, (10 items) and (d) time management (5 items). Most of the items are scored Can (1) or Cannot (0), and three items are scored Can (2), On the way (1), or Cannot (0). The new version was validated with Rasch analyses showing acceptable psychometric properties [12], which enabled transformation of ordinal raw total scores of the KaTid-Senior into interval measures [25].

#### 2.2.3. Assessments to Measure DTM

Time-S© is an instrument for self-rating DTM that has been validated for adults with mental and neurodevelopmental disabilities [26]. Time-S has been adapted and validated for older adults with mild to moderate dementia (Time-S Senior) by removing non-relevant items, e.g., items specific to adults in working age [12]. Time-S Senior contains 17 items with a Likert-like frequency scale with four response alternatives ranging from Never (1) to Always (4).

Time-Proxy© is an instrument used for proxy-rating of a person’s DTM. Previously, Time-Proxy has been used for youth with physical and intellectual disabilities [24,27]. The questions in Time-Proxy largely correspond to the questions in Time-S. In this study, significant others’ ratings of the PwDs’ DTM were made in an adjusted and validated version of Time-Proxy [12]. This version encompasses 10 items with five rating options: four ranging from Never (1) to Always (4), along with “I don’t know.” “I don’t know” was treated as a missing value.

#### 2.2.4. Approximation Level of Dementia

The MMSE is a widely used screening test of cognitive functions [13]. In this study, the Swedish version of the MMSE was used to estimate cognitive level in persons with dementia [28]. The MMSE, with a total score of 30, includes the following sub-items: orientation (10), registration (3), attention and calculation (5), recall (3), language (8), and figure copying (1).

### 2.3. Procedure

During 2018–2021, participants were recruited from ten memory clinics in ten different regions in Sweden. Twelve registered occupational therapists working at the memory clinics conducted the recruitment and data collection using the demographic questionnaire, KaTid-Senior form, and Time-S Senior form for PwDs. The assessments were carried out in clinical settings or in the PwDs’ homes. At the same time, a significant other of the PwD answered the demographic questionnaire and the Time-Proxy form.

Before the data collection started, all occupational therapists on the memory clinics were trained in using the instruments. Data on dementia diagnoses and MMSE results were retrieved from medical records.

### 2.4. Statistical Analyses

A power analysis with the intent to use a multiple regression analysis including three independent variables and an assumption of an adjusted R square of 20% showed that a group of at least 36 persons is needed in the study to have 80% power and 5% significance level.

Depending on the results of normal distribution testing, Pearson’s r or Spearman’s rho were used to examine bivariate associations between measurements and *t*-tests for controlling for gender differences for potential associations between outcomes and demographic data. The calculations were made with IBM SPSS Statistics for Windows, Version 27.0. Armonk, NY, USA.

To further explore the association between TPA and dementia severity, MMSE sub-items correlating with the KaTid-Senior with r ≥0.30 were selected for multiple linear regression analysis. Prior to the regression analyses, analyses to ensure that there were no violations of the assumptions of linearity, independence, normality, and equal variance were conducted. Any possible impact of outliers was evaluated for each model by removing outliers before running the regression analysis again, and then comparing models. Adjusted R2 was used to evaluate model fit. The level of significance was set at *p* < 0.05. Statistical analyses were calculated with IBM SPSS Statistics for Windows, Version 27.0. Armonk, NY, USA. No adjustment to experimental alpha was made for multiple testing, and therefore, caution should be added for the risk of inflated Type I error rate.

## 3. Results

### 3.1. Characteristics of Participants

The gender distribution was relatively even for PwDs (47.2% women, 52.8% men), while a small majority of SOs were women (57.1%). The mean age was 74.9 years for PwDs and 67.1 years for SOs. Alzheimer’s disease was the most common dementia diagnosis (71.7%) and the mean result for PwDs on the MMSE was 22.4 out of 30. The majority of PwDs were living together with someone (81.1%), and most of the SOs were spouses (83.7%). Many SOs (71.4%) provided daily support to the person with dementia (Table 1).

### 3.2. Bivariate Associations between TPA and DTM and Severity of Dementia

The bivariate analyses presented in Table 2 showed a significant positive correlation between TPA (KaTid-Senior) and the dementia severity (MMSE); that is, the more severe dementia, the lower the TPA. In addition, analyses of total TPA in relation to MMSE sub-items showed significant weak correlations between TPA and orientation, attention and calculation, and language, and a moderate correlation between TPA and figure copying. Further bivariate analyses between the KaTid-Senior subgroups and the MMSE sub-items showed significant correlations in the following: time orientation/time concepts correlated with orientation, recall, and figure copying; time orientation/objective time correlated with attention and calculation, language, and figure copying; and time management correlated with attention and calculation, and figure copying.

No significant correlation was found between TPA (KaTid-Senior) and self-rated DTM (Time-S Senior), but significant correlations were found between TPA and proxy-rated DTM (Time-Proxy), and between proxy-rated DTM and self-rated DTM. Proxy-rated DTM also correlated with dementia severity (MMSE). No associations were found between either the TPA, the self-rated DTM, or the proxy-rated DTM and demographic data such as age, gender, education, or support given (not presented in Table 2).

### 3.3. Association between TPA and Cognitive Functions

As shown in Table 3, in the multiple regression model the MMSE sub-items, orientation, attention and calculation, and, particularly, figure copying were significant predictors of TPA (dependent variable). One outlier was removed to improve the model.

### 3.4. Associations between TPA Subcategories and Cognitive Functions

Three multiple linear regression analyses between the dependent TPA variables time orientation/time concepts, time orientation/objective time, and time management (KaTid-Senior subgroups), and the independent cognitive variables orientation, attention and calculation, and figure copying (MMSE sub-items), revealed several significant correlations as reported in Table 4. Figure copying was the most significant predictor of all TPA variables: time orientation/time concepts, time orientation/objective time, and time management. The sub-item attention and calculation was significant in predicting time orientation/objective time and time management. Omitting occasional outliers only slightly improved models and, therefore, the results reported in Table 4 include outliers.

## 4. Discussion

The aim of this study was to investigate the associations between TPA and DTM and dementia severity. There were significant correlations between TPA and dementia severity, especially with visuospatial functions. Significant correlations were also found between proxy-rated DTM and TPA, dementia severity, and PwDs’ self-ratings of DTM. However, no significant correlations were found between the PwDs’ self-rated DTM and TPA or dementia severity.

By revealing associations between all three levels of TPA and dementia severity, this study adds knowledge as to how underlying time functions are related to dementia severity. Previous research has shown that time orientation functions and spatial functions are connected to the hippocampus [29] and are processed in the right posterior parietal cortex, which is often affected by dementia [29,30]. Correspondingly, a significant moderate correlation was found between TPA and the figure copying—a function that is dependent on good visuospatial ability. Figure copying was also found to be associated with time orientation/time concepts, time orientation/objective time, and time management. This indicates that spatial functions are necessary for persons with dementia to be able to process time at all levels. The correlation between objective time/time concepts and the MMSE sub-item orientation could also be expected as both involve time orientation. Correlations between time orientation/objective time, time management, and MMSE sub-items attention and calculation could be anticipated as well, as previous research has suggested that short-term memory and attention are required for time estimation in persons with Alzheimer’s disease [31]. Importantly, the strongest correlations for the sub-item figure copying, as well as attention and calculation, were related to the higher levels of TPA.

However, the MMSE and other cognitive tests are frequently used for supporting diagnostics [2,3] and not for further rehabilitation planning or efforts to support daily living. Our findings suggest the need for in-depth TPA and DTM assessments at an early stage of dementia, especially when visuospatial or attentional impairments are observed. A previous study revealing associations between MMSE sub-items and activities of daily living (ADL) for patients with Alzheimer’s disease suggested that this could be useful information for clinicians, enabling early detection of patients in need of ADL support [32]. Similar assumptions could be made from the association between the MMSE and TPA. Early detection of TPA impairments is also advantageous since the timing of interventions, such as early implementation of time assistive technologies or compensatory strategies to enable time-dependent occupations, is of utmost importance as declining cognitive skills can make it too difficult to learn how to use new strategies or devices [7]. Research has shown that when the degree of cognitive impairment increases, older adults become less able to implement compensatory strategies [33]. As dementia is a progressive disease, it is important for health care professionals to be able to provide early interventions when the PwDs are most likely to benefit from them. However, MMSE results cannot, on their own, be used to predict TPA and DTM impairments, but rather indicate a need for an extended assessment of TPA and DTM—especially if failure on the visuospatial sub-item (figure copying) is observed. Further research is needed to confirm these findings.

Although this study found no significant associations between self-rated DTM (Time-Senior), TPA (KaTid-Senior), and dementia severity (MMSE), it is important to consider a PwDs’ own perception of their DTM. A significant association between TPA (KaTid-Senior) and self-rated DTM (Time-S Senior) might have been expected because these two concepts are suggested to be related [22]. One conclusion is that KaTid-Senior and Time-S Senior measure different aspects of time. Another explanation might be the discrepancy between an objective measure (KaTid-Senior) and self-ratings (Time-S), which is in accordance with research, suggesting that objective measurements do not always correspond with the individual’s experiences or opinions [15,34,35]. There is also a risk of response bias in cognitive self-ratings due to, for example, impaired memory functions, emotional responses on sensitive questions, or overestimation of one’s ability [36,37]. Another important factor is that impaired awareness of dementia and its consequences is common and has been also observed in mild stages of Alzheimer’s disease [38], even if persons with mild and moderate dementia have been found to be partially aware of their disabilities and able to rate their everyday functioning with reasonable accuracy [38,39,40]. Moreover, this study found a significant association of medium effect size between self-reported DTM in Time-S Senior and proxy-rated DTM in Time-Proxy. Thus, it is important to consider PwDs’ perceptions and experiences of their DTM, and to evaluate awareness of problems individually. Using self-ratings along with objective measures and proxy-ratings can help health care professionals in understanding a PwDs’ own conceptions of their DTM and form a basis for dialogue when planning interventions to enhance occupational performance.

Proxy ratings are widely used in research and clinical settings and this study emphasises the importance of information provided by significant others. According to the results, there was not only a significant association between proxy-rated DTM (Time-Proxy) and self-rated DTM (Time-S Senior), but also significant associations between proxy-rated DTM and TPA (KaTid-Senior), time orientation/time concepts and time orientation/objective time (KaTid subgroups), dementia severity (MMSE), and the MMSE sub-item orientation. The associations between proxy-rated DTM and time orientation functions may be because impairments in orientation to time often lead to repeated questions about time and dates and, therefore, become extra apparent, while impairments in experience of time may be more difficult to notice. Time management impairments might not be discovered if, for example, a partner has the main responsibility for time planning. Thus, there are reasons to combine proxy-ratings with self-ratings and observational assessments to get a comprehensive overview of a PwD’s abilities. Moreover, earlier research has shown that proxy-ratings tend to underestimate the functional ability of PwDs, indicating that over-reliance on proxy-ratings in research and clinical settings may be problematic [41]. Noteworthy, there are also previous studies that did not find significant correlations between proxy ratings and self-ratings [38,41], while another study found a significant correlation between TPA and proxy-rated DTM, but no significant correlation between TPA and self-rated DTM [10]. Hence, relying on proxy ratings or self-ratings alone may be inadvisable, thus suggesting that they should be used in combination to get a comprehensive picture of a PwD’s DTM. Taken together, the toolbox for assessment of time functions and skills should include both observational instruments on a PwDs’ TPA on a functional level, such as KaTid-Senior, as well as self-ratings and proxy ratings of DTM that add more in-depth knowledge and give a broader picture on an activity level. For a client-centred approach, self-ratings and proxy ratings of DTM are important complements that should be considered in the light of the PwD’s awareness of the consequences of dementia and within the experience of significant others, especially in the context of caregiving relationships.

## 5. Study Limitations and Implications for Future Research

Even though the power estimation showed that a sample of at least 36 participants would be sufficient for the planned analyses, a larger sample size might have provided more reliable estimates and the results should therefore be interpreted with caution. However, the COVID-19 pandemic significantly reduced the possibility for the memory clinics to recruit new participants.

Further investigation is warranted, for example, by including comparisons of TPA across different types of dementia. Interpretation of occasional outliers in the current analyses indicated that there might be differences between different dementia diagnoses, but there were not enough data to be able to draw such conclusions. In future research, it would also be of interest to explore changes in PwDs’ TPA and DTM over time and in relation to interventions. Moreover, the sample in this study included PwDs ≥60 years. Research has shown that there are differences in cognitive functioning between persons with early-onset and late-onset dementia [42]; therefore, results might not be generalised across age groups.

## 6. Conclusions

The correlations between TPA and dementia severity, and TPA and visuospatial abilities, are in line with previous research on the association between spatial functions and time-related functions. This study adds that, when visuospatial impairments are observed, it could also be a sign of TPA impairments, which requires further investigation of TPA and DTM—preferably at an early stage of dementia. Likewise, attentional deficits could be an indication of TPA impairments. For a comprehensive assessment of TPA and DTM, objective measures should be used in combination with self-ratings and proxy ratings as a basis for individually-adapted intervention planning. The results from this study can be used in further development of methods to identify and compensate for impaired TPA and support DTM in PwDs.

## Figures and Tables

**Table 1:** Basic characteristics of participants with dementia and their significant others.

Persons with Dementia (*n* = 53)		
Gender *n* (%)	Women	25 (47.2)
	Men	28 (52.8)
Age, years (mean, SD)		74.9 (6.9)
Diagnosis *n* (%)	Alzheimer’s disease	38 (71.7)
	Vascular dementia	6 (11.3)
	Dementia with Lewy bodies	4 (7.5)
	Dementia (other)	3 (5.7)
	Alcohol dementia	1 (1.9)
MMSE (mean, SD)		22.4 (4.0)
Family situation *n* (%)	Single, living alone	7 (13.2)
	Partner, not living together	3 (5.7)
	Partner/family, living together	43 (81.1)
Education *n* (%)	Elementary school	22 (41.5)
	High school	14 (26.4)
	College/University	16 (302.2)
	Missing information	1 (1.9)
Significant others (*n* = 49)		
Gender *n* (%)	Women	28 (57.1)
	Men	21 (42.9)
Age, years (mean, SD)		67.1 (9.7)
Relation to participant *n* (%)	Spouse	41 (83.7)
	Child	7 (14.3)
	Missing information	1 (2.0)
Support given (days/week) *n* (%)	≤1	6 (12.2)
	2–3	6 (12.2)
	Daily	35 (71.4)
	Missing information	2 (4.1)

**Table 2:** Bivariate associations between time processing ability (KaTid-Senior), daily time management (Time-S Senior), proxy daily time management (Time-Proxy), and dementia severity (MMSE).

Variables	KaTid-Senior					Time-S Senior	Time-Proxy
	Total Score (Logits)	A Time Perception/Time Sense	B Time Orientation/Time Concepts	C Time Orientation/Objective Time	D Time Management		
Time-S Senior (logits)	0.132	−0.049	0.164	0.112	0.127		
Time-Proxy (logits)	0.381 ** ^1^	0.225	0.398 **	0.323 *	0.217	0.363 *	
MMSE total score	0.359 **^1^	0.212	0.420 **	0.500 **	0.491 **	0.209	0.325 * ^1^
MMSE orientation	0.315 *	0.196	0.330 *	0.204	0.254	0.222	0.301 *
MMSE registration	0.037	−0.173	−0.032	0.116	0.023	0.053	0.118
MMSE attention, calculation	0.367 **	−0.010	0.209	0.373 **	0.462 **	0.072	0.133
MMSE recall	0.243	0.238	0.315 *	0.123	0.133	0.045	0.137
MMSE language	0.296 *	0.216	0.173	0.295 *	0.211	0.170	0.256
MMSE figure copying	0.507 **	0.163	0.366 **	0.462 **	0.577 **	0.077	0.244

^1^ Correlations performed with Pearson’s r. All other correlations were performed with Spearman’s rho. ** *p* < 0.01; * *p* < 0.05.

**Table 3:** Multiple linear regression model of time processing ability (KaTid-Senior) to orientation, attention and calculation, and figure copying (MMSE sub-items).

Dependent TPA Variable	Independent Cognitive Variables	B	95% CI		*p*-Value	*p*-Value (Sig.), R^2^ and Adjusted R^2^ for the Model
			Lower Bound	Upper Bound		
KaTid-Senior	MMSE Orientation	0.236	0.000	0.471	0.050	*p* < 0.001
	MMSE Attention, calculation	0.197	0.003	0.392	0.047	R^2^ = 0.407
	MMSE Figure copying	1.180	0.470	1.890	0.002	Adjusted R^2^ = 0.368

Abbreviations: TPA = time processing ability, B = regression coefficient, CI = confidence interval.

**Table 4:** Multiple linear regression models of time orientation/time concepts (KaTid-Senior part B), time orientation/objective time (KaTid-Senior part C), and time management (KaTid-Senior part D), respectively, to orientation, attention and calculation, and figure copying (MMSE sub-items).

Dependent TPA Variables (KaTid-SENIOR Subcategories)	Independent Cognitive Variables	B	95% CI		*p*-Value	*p*-Value (Sig.), R^2^ and Adjusted R^2^ for the Model
			Lower Bound	Upper Bound		
Time orientation/ time concepts	MMSE Orientation	0.372	0.087	0.657	0.012	*p* = 0.001 R^2^ = 0.291 Adjusted R^2^ = 0.245
	MMSE Attention, calculation	0.082	−0.150	0.313	0.482	
	MMSE Figure copying	1.074	0.225	1.923	0.014	
Time orientation/ objective time	MMSE Orientation	0.092	−0.327	0.510	0.662	*p* < 0.001 R^2^ = 0.345 Adjusted R^2^ = 0.302
	MMSE Attention, calculation	0.464	0.123	0.804	0.009	
	MMSE Figure copying	1.732	0.485	2.979	0.008	
Time management	MMSE Orientation	0.169	−0.063	0.402	0.149	*p* < 0.001 R^2^ = 0.460 Adjusted R^2^ = 0.424
	MMSE Attention, calculation	0.226	0.037	0.415	0.020	
	MMSE Figure copying	1.435	0.744	2.126	<0.001	

Abbreviations: TPA = time processing ability, B = regression coefficient, CI = confidence interval.

## Data Availability

Anonymized data can be available from the authors on reasonable request after all the planned manuscripts for the project “Managing Time with Dementia” have been published.
